# Reviewed article: Mattos CDCL, Tomazelli J, Girianelli VR. Trends and characteristics of congenital syphilis in children under 1 year of age: a time series analysis, municipality of Rio de Janeiro, 2007-2021. Epidemiol Serv Saude. 2026:35;e20250109

**DOI:** 10.1590/S2237-96222026v35e20250109.c

**Published:** 2026-04-17

**Authors:** Sandra Maria do Valle Leone de Oliveira

**Affiliations:** 1 Fundação Oswaldo Cruz, Campo Grande, MS, Brazil

## First round comments

O artigo está apto para publicação, considerando sua pertinência, metodologia adequada e potencial contribuição científica para o tema de vigilância em saúde e política pública no âmbito da epidemiologia.

O manuscrito é relevante, atual, bem estruturado e oferece contribuições concretas à compreensão da sífilis congênita no cenário urbano brasileiro. O uso de métodos robustos e o recorte territorial aprofundado qualificam ainda mais os achados.

Entretanto alguns ajustes são necessários:

1- Reforçar a discussão sobre subnotificação por tipo de serviço (público x privado);

2-Incluir brevemente outras possíveis fontes de variação territorial (ex: deslocamento para parto, distribuição de equipes de APS);

3- Avaliar a possibilidade de mencionar o uso de médias móveis como técnica complementar (mesmo que não utilizada), reforçando a escolha metodológica pelo joinpoint.

4- Revisar a frase : Acesso aos dados e métodos de limpeza

Realizou-se download dos dados do SINAN e SINASC disponibilizados na página do TABNET do Município do Rio de Janeiro, em maio de 2022. O acesso aos dados deve seguir a recomendação da revista. Nesse tópico não foram mencionados os métodos de limpeza. Informar corretamente a disponibilidade dos dados é requisito de transparência e integridade em pesquisa, previstos nas Política editorial (https://ress.iec.gov.br/p/page/0/peditorial) e instruções aos autores da nossa revista (https://ress.iec.gov.br/p/page/2/instrucoes), e que reproduzimos a seguir:

Política editorial: “Os dados de pesquisa (como bancos de dados, códigos, métodos e outros materiais utilizados ou resultantes da pesquisa) devem, preferencialmente, ser depositados em repositórios de dados confiáveis, como o SciELO Data (lista disponível em https://wp.scielo.org/wp-content/uploads/Lista-de-Repositorios-Recomendados_pt.pdf), com um identificador persistente, como o DOI (Digital Object Identifier).

O acesso a esses dados, por meio de um link para o repositório, deve ser informado na seção “Disponibilidade de dados” e a referência completa deve ser incluída na seção de “Referências”, conforme exemplo abaixo:

Andrade M. Estudo de genes em ratos albinos na América Latina. OSF [dataset], 2018, ASM0000v1. http://dx.doi.org/10.1590/0123-45620187214”

Instruções: “Disponibilidade dos dados do artigo: declaração sobre o acesso aos dados de pesquisa (bancos de dados, códigos, métodos e outros materiais utilizados e resultantes da pesquisa), informar link do repositório e referenciamento, com a devida citação no texto”

Recomendação da revista para acesso aos dados:

5-Recomenda-se que o manuscrito explore os impactos potenciais da incompletude dessa variável sobre os achados, diferenciando entre inviabilização total e limitação interpretativa. Além disso, seria pertinente discutir estratégias metodológicas ou análises de sensibilidade que poderiam ser adotadas em estudos futuros para contornar ou compensar dados faltantes - como imputações, análises estratificadas pelos anos com menor percentual de dados ignorados, ou até triangulação com outras fontes. 

Recommendation: Major revision

## Second round comments

O artigo é relevante, apresenta rigor metodológico e contribui para discussão da Sífilis. Entre as minhas preocupações:

1-Reduzir repetições excessivas e consolidar dados no texto principal. Ex. “As taxas entre mães brancas foram as mais baixas, variando de 6,1/mil em 2011 a 6,8 em 2021, com maior taxa em 2014 (9,5)...”

Sugestão : “As taxas entre mães brancas mantiveram-se mais baixas e estáveis ao longo do período (p=0,647), conforme evidenciado na Figura 4.” As taxas ano a ano estão no gráfico e não precisam ser repetidas no corpo do texto, a menos que se queira destacar uma mudança abrupta.

Outro exemplo: o parágrafo “A 27ª superou também o município em 2008 (13,9), 2019 (28,4) e 2020 (28,3) e a 5ª de 2018 (16,4) a 2020 (19,1). Nas 4ª e 6ª as taxas estavam abaixo da área, sendo maiores em 2021, 5,6 e 9,0, respectivamente. Todas as regiões administrativas tiveram tendência crescente (APC≥6,6; p-valor≤ 0,024), apesar de flutuação, principalmente a 27ª RA.” Nesse caso, pode-se eliminar a redundância e números específicos pouco relevantes para a interpretação geral. Os valores podem permanecer em figuras/tabelas e ser sumarizados no texto. Ex. Na área de planejamento 2.1, as regiões da Rocinha (27ª RA) e da 5ª RA apresentaram taxas superiores ao município em vários anos, com tendência crescente estatisticamente significativa (APC≥6,6; p≤0,024), apesar de flutuações, especialmente na 27ª.”

2-Qualificar com mais cautela as inferências relacionadas ao impacto da atenção básica e da pandemia. Sugere-se explicitar na discussão que essas associações são hipóteses interpretativas e não inferências causais confirmadas estatisticamente. Essas hipóteses devem ser retiradas das conclusões do resumo e do texto principal, limitando-se a responder os resultados encontrados apartir dos objetivos.

3-Reforçar na discussão a distinção entre taxas brutas e ajustadas e a importância da análise de tendência estatisticamente significativa.Este ponto é importante e poderia ser melhor enfatizado na discussão, como evidência da relevância de controlar a análise pela base populacional, especialmente diante da redução dos nascimentos no município.

4- Ampliar a problematização da queda entre mães pretas e o persistente alto nível de desigualdade.

Recommendation: Minor revision

